# Reforestation-induced aerosol cooling effects divergently modulated by various types of biogeophysical feedback

**DOI:** 10.1093/nsr/nwaf323

**Published:** 2025-08-15

**Authors:** Jialei Zhu, Joyce E Penner, Hao Liu, Qinghao Guo, Yaxin Liu, Junjun Deng, Xi Zhao, Cong-Qiang Liu, Pingqing Fu

**Affiliations:** Institute of Surface-Earth System Science, School of Earth System Science, Tianjin University, Tianjin 300072, China; Department of Climate and Space Sciences and Engineering, University of Michigan, Ann Arbor, MI 48109, USA; Institute of Surface-Earth System Science, School of Earth System Science, Tianjin University, Tianjin 300072, China; Institute of Surface-Earth System Science, School of Earth System Science, Tianjin University, Tianjin 300072, China; Institute of Surface-Earth System Science, School of Earth System Science, Tianjin University, Tianjin 300072, China; Institute of Surface-Earth System Science, School of Earth System Science, Tianjin University, Tianjin 300072, China; Institute of Surface-Earth System Science, School of Earth System Science, Tianjin University, Tianjin 300072, China; Institute of Surface-Earth System Science, School of Earth System Science, Tianjin University, Tianjin 300072, China; Institute of Surface-Earth System Science, School of Earth System Science, Tianjin University, Tianjin 300072, China

**Keywords:** reforestation and afforestation, biogeophysical effects, biogenic secondary organic aerosols, radiative effects, feedback pathways

## Abstract

Reforestation and afforestation alter climate not only through biogeophysical processes such as changes in surface albedo, evapotranspiration and near‐surface turbulence, but also by modifying emissions of biogenic volatile organic compounds (BVOCs) that drive biogenic secondary organic aerosol (BSOA) formation. Using an Earth system model coupled with an advanced aerosol module, we quantify how biogeophysical feedback from vegetation change influences BVOC emissions, BSOA burden and aerosol radiative effects under future land‑use scenarios. Our results reveal that biogeophysical feedback either amplifies or offsets BSOA cooling, depending on regional climate–vegetation interactions. In regions where reduced surface albedo dominates, increasing temperature and BVOC emissions enhance BSOA burden and its radiative cooling. Conversely, in regions where updrafts and cloud formation are enhanced, reduced surface radiation suppresses BVOC emissions and offsets BSOA increases from vegetation changes alone. Globally, these types of feedback amplify BVOC emission changes in 52% of reforested areas but suppress them elsewhere, intensifying spatial heterogeneity in aerosol climate effects. These divergent feedback pathways introduce strong spatial heterogeneity and non-linearity into the BSOA–climate response. Incorporating such biogeophysical modulation of BSOAs is essential for designing reforestation strategies that maximize climate mitigation benefits.

## Teaser

Reforestation can amplify or diminish its induced aerosol cooling effects through biogeophysical feedback modulation, driving strong regional variation in net climate benefits.

## INTRODUCTION

Reforestation and afforestation have emerged as key strategies for mitigating climate change [1–3], while also being recognized as important natural climate solutions [4–6]. However, these land-use changes trigger complex biogeophysical effects that alter surface energy and water cycles [7], which results in various climate and environmental impacts and connects changes across the atmosphere, soil and biosphere [8,9]. The feedback mechanisms between these components of the Earth system further complicate the assessment and prediction of global climate changes driven by changes in vegetative cover [10], potentially impacting the maximization of the cooling effects of reforestation and afforestation actions outlined in the United Nations 2030 Sustainable Development Goals (SDG 15.2).

Changes in vegetation can trigger a range of climate effects through various biogeophysical processes, with the net impact hinging on the interplay between warming and cooling influences [11–13]. Forests modify surface properties compared to other vegetation types by their low albedo reducing shortwave radiation reflection [11,14], which leads to a warming effect [15–18]. On the flip side, forests boast higher evapotranspiration rates than croplands [19]. The evaporation of water from the large leaves of forest plants absorbs heat, thereby cooling the surrounding air [20]. This cooling effect is especially pronounced in low-latitude regions where evapotranspiration is more intense [21]. Additionally, vegetation cover shapes surface sensible and latent heat fluxes, with reforestation and afforestation contributing to an estimated cooling effect [7,22]. Moreover, forests have higher surface roughness compared to croplands, enhancing near-surface turbulence [12]. This turbulence promotes the exchange of heat, momentum and water vapor between the surface and the atmosphere [20,23]. Increased updrafts can also foster the formation of low-level convective clouds, which produce a cooling effect [15,24]. The biogeophysical changes in vegetation also reshape atmospheric circulation patterns [25–27], potentially sparking both global and regional climate shifts [28,29]. Thus, changes in vegetation can influence atmospheric circulation, radiation, temperature and other meteorological conditions through a variety of intricate biogeophysical feedback processes.

A critical but poorly understood aspect of these biogeophysical effects involves their influence on biogenic volatile organic compound (BVOC) emissions from plants and subsequent formation of biogenic secondary organic aerosols (BSOAs) [30,31]. Biogenic secondary organic aerosols can constitute 15%–80% of global atmospheric fine particulate mass [32], affecting climate by scattering or absorbing solar radiation and acting as cloud condensation nuclei [33–36]. Forests, especially broadleaf and needleleaf types, emit more BVOCs than crops, so reforestation and afforestation increase BVOC emission [37], while deforestation decreases it [31,38]. The emission rates also vary with climate, as high temperature and increased radiation usually promote BVOC emission [39]. The net impact of reforestation-induced biogeophysical feedback on BSOA formation and its radiative forcing remains unquantified. Studies using site and satellite observations, as well as simulations, have examined the feedback mechanisms between BVOC emission and aerosol–cloud interactions [40,41]. Current assessments using fully coupled models struggle to differentiate between the impacts of vegetation changes and climate change induced by biogeophysical feedback on BVOC emission, while offline models often overlook climate feedback, creating uncertainties [31]. Accurate assessment of how vegetation changes affect BVOC emission and climate requires quantification of the contribution from biogeophysical feedback.

How biogeophysical feedback from reforestation and afforestation modulates BVOC emissions and BSOA radiative effects remains unresolved. Here, we address this gap by isolating biogeophysical feedback within the Earth system model framework under harmonized future land‑use scenarios, quantifying the individual influences on BVOC emissions, BSOA burden and aerosol radiative forcing. We reveal that the biogeophysical feedback induced by reforestation would act in opposite directions for BVOC emission depending on dominant feedback mechanisms, which either offset or enhance the radiative effects of BSOAs resulting from vegetation changes. Our result suggests that various types of biogeophysical feedback on BVOC emission and the subsequent BSOA radiative effects should be included in the assessment and prediction of climate change induced by vegetation changes.

## RESULTS AND DISCUSSION

### Biogeophysical effects dominated by decreased albedo increase BSOA formation

**Figure 1.**
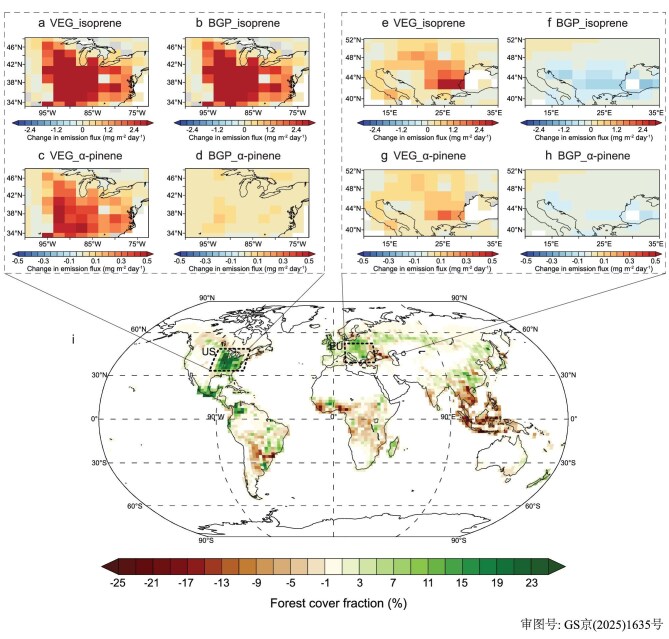
Predicted changes in global forest cover and BVOC emission intensities in typical afforestation regions. (a–d) Changes in isoprene (a and b) and α-pinene (c and d) emission intensities (mg m⁻² day⁻¹) for the US region. (e–h) Changes in isoprene (e and f) and α-pinene (g and h) emission intensities (mg m⁻² day⁻¹) for the EU region. (a, c, e and g) Changes are attributed to vegetation-type change effect (VEG effect), which are differences solely due to projected shifts in vegetation types. (b, d, f and h) Changes are attributed to biogeophysical feedback effect (BGP effect), which are differences arising from climate feedback induced by vegetation changes. (i) Global distribution of projected forest cover fraction changes (2000–2100) under the SSP245 scenario. Dashed boxes highlight the two key afforestation regions analyzed: eastern US and central-western Europe (EU).

Vegetation cover continues to change due to both natural variations and human activities aimed at meeting increased food demands. Since industrialization, global vegetation has been extensively converted to cropland to meet increased food demands. However, according to the SSP245 scenario, there will be a shift during the 21^st^ century in relatively developed regions such as the USA, Europe and East Asia, where reforestation efforts will gradually increase forest replacing other vegetations (e.g. crop, grass, shrub) to optimize the living environment, while much of the other regions will continue to expand cropland (Fig. 1i) [42,43]. The biogeophysical effects (e.g. changes in albedo, surface roughness, evapotranspiration) of vegetation change can induce regional and global climate change. This alteration, in turn, affects the emission of BVOCs, leading to changes in the formation of BSOAs and their radiative effects indirectly. These effects are referred to as BGP (biogeophysical) effects in this study. Here, we first evaluated the BGP effects in the eastern US and central-western Europe (EU), which are the largest afforested areas under the SSP245 scenario from 2000 to 2100 (regions shown by dashed boxes in Fig. 1i).

**Figure 2.**
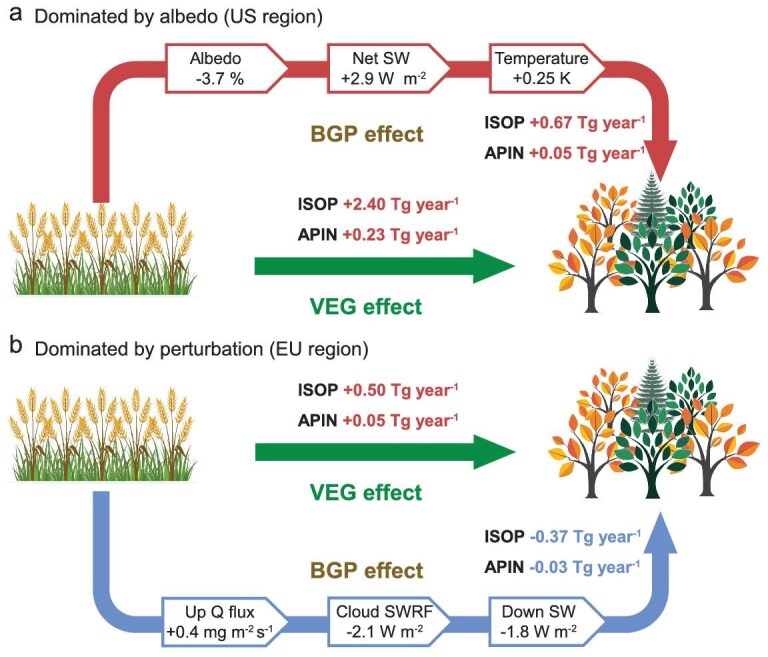
Mechanisms driving afforestation-induced changes in BVOC emissions for the US and EU regions. Schematic diagrams illustrate the dominant biogeophysical feedback pathways and their consequences for isoprene (ISOP) and α-pinene (APIN) emissions in the US (a) and EU (b) regions (dashed boxes in Fig. 1). (a) US region (net emission increase): afforestation reduces surface albedo, leading to increased net shortwave radiation (Net SW) absorption and higher surface air temperatures. This warming effect amplifies the increase in BVOC emissions initially driven by vegetation-type change (VEG effect) through the biogeophysical feedback (BGP effect). (b) EU region (net emission suppression): afforestation enhances upward airflow and moisture flux (Up Q Flux), promoting cloud formation. This increases negative cloud shortwave radiation forcing (Cloud SWRF), reducing downward shortwave radiation (Down SW) at the surface. The resultant cooling offsets most of the increase in BVOC emissions driven by the VEG effect.

During the reforestation process in the US region, deciduous broadleaf forests (∼0.37 Mkm²) and evergreen needleleaf forests (∼0.09 Mkm²) will be planted (Supplementary data, Fig. S1). The conversion of cropland to forest results in a decrease in surface albedo of ∼3.7% (Fig. S2a), leading to an increase in average surface net shortwave radiative flux by 2.9 W m⁻² in the summer (Fig. S3a), which is the crucial season of vegetation growth and BVOC emission in the middle latitudes of the Northern Hemisphere. Although reforestation leads to an increase in sensible and latent heat fluxes by 1.5 W m⁻² in the US region (Fig. S4a), increase in net shortwave radiative flux (2.9 W m⁻²) predominates, driving an increase in the average 2 m and surface air temperature in the summer by 0.25 and 0.22 K respectively (Fig. S5a and b). Moreover, the dynamic perturbation (i.e. change in local atmospheric circulation pattern) induced by vegetation change dominates over thermodynamic effects in the variation in vertical air motion, resulting in enhanced subsidence during summer in the US region (Fig. S6a), leading to a reduction in cloud cover and a decrease in total cloud water path by 2.1 g m⁻² (Fig. S7a). This leads to a less negative cloud shortwave radiative forcing by 1.7 W m⁻² (Fig. S8a), resulting in an increase in downward shortwave radiative flux at the surface by 2.6 W m⁻² in the summer (Fig. S9a). The increase in surface temperature and shortwave radiative flux due to changes in albedo from reforestation promotes the emission of BVOCs (Fig. 1a and c). When only the change in vegetation types (VEG effects) are considered, isoprene emissions in the US region increase from 7.92 to 10.32 Tg year⁻¹ (an increase of 30%) from 2000 to 2100, and α-pinene emission increases from 1.06 to 1.29 Tg year⁻¹ (an increase of 21%) (Fig. 1a and c). When accounting for the feedback impacts of temperature and radiation changes due to the BGP effects on BVOC emission, isoprene emissions further increase by 0.67 Tg year⁻¹ to 11.02 Tg year⁻¹, equivalent to amplifying the changes attributed to VEG effects by 28% (Figs 1b and 2a). Similarly, α-pinene emission increases by an additional 0.05 Tg year⁻¹ to 1.34 Tg year⁻¹, corresponding to a 22% increase due to VEG effects (Figs 1d and 2a). The increase in the emissions of BVOCs leads to an increase in the burden of BSOAs. The annual average BSOA concentration in the US region increases by 175 μg m⁻² (a 10% increase) due to VEG effects of reforestation in the US region (Fig. S10a), while the BGP effects further increase the annual average BSOA burden by an additional 28% (50 μg m⁻²) (Fig. S10b), bringing the total increase to 13% (Fig. S10c).

### Biogeophysical effects dominated by enhanced updraft decrease BSOA formation

Unlike the US region, the simulation indicates a decrease in both surface air temperature and the downward shortwave radiative flux after reforestation in the EU region (Figs S5d and S9b). From 2000 to 2100, the EU region will increase evergreen needleleaf forests by ∼0.12 Mkm², deciduous broadleaf forests by ∼0.15 Mkm², and grasslands by ∼0.09 Mkm² while decreasing cropland (Fig. S11), resulting in a 2% decrease in surface albedo (Fig. S2b). Meanwhile, turbulence perturbations caused by vegetation changes enhance upward airflow in the EU region in the summer (Fig. S6b), which is in contrast to the downward airflow perturbations in the US region (Fig. S6a). The enhanced updraft in the EU region results in a 0.4 mg m⁻² s⁻¹ increase in the upward water vapor flux from the surface in the summer (Fig. S12b), promoting localized cloud development and increasing cloud water path by 1.3 g m⁻² (Fig. S7b). The enhanced albedo of clouds results in a 2.1 W m⁻² more negative cloud shortwave radiative forcing in the EU region (Fig. S8b), leading to a decrease in shortwave radiative flux reaching the surface by 1.8 W m⁻² (Fig. S9b). Despite the decrease in surface albedo, the average net shortwave radiative flux at the surface decreases by 0.9 W m⁻² during summer (Fig. S3b). Similar to the US region, reforestation leads to an increase of 1.4 W m⁻² in sensible and latent heat fluxes averaged over the summer (Fig. S4b). The combined decrease in net shortwave radiation and increase in sensible and latent heat fluxes result in a decrease of 0.34 and 0.35 K in the summer average 2 m air temperature and surface temperature in the EU region, respectively (Fig. S5c). The decrease in downwelling radiation and temperature together contribute to a reduction in BVOC emission in the EU region (Fig. 2b). The annual isoprene emission in the EU region is increased by 0.50 Tg year⁻¹ due to VEG effects of reforestation (from 5.16 to 5.66 Tg year⁻¹, an increase of ∼10%) (Fig. 1e), while the BGP effects lead to a 75% offset in the emission increment (annual average emission of 5.29 Tg year⁻¹, a decrease of 0.37 Tg year⁻¹) (Figs 1f and 2b). The increase in α-pinene emissions in the EU region is also suppressed by 60% (0.06 Tg year⁻¹) due to the BGP effects (Figs 1h and 2b). The changes in isoprene and α-pinene emissions in the summer are over 80% of the annual average changes. While the VEG effects increase the annual average BSOA concentration in the EU region by 82 μg m⁻² (Fig. S13a), the BGP effects decrease it by 47 μg m⁻² (Fig. S13b), offsetting 58% of the BSOA concentration increase caused by only the difference in vegetation.

Comparing the responses of BVOC emission to BGP effects induced by vegetation changes in the US and EU regions reveals that differences in the dominant physical processes may lead to disparities in how vegetation changes contribute to regional BVOC emission and BSOA concentrations (Fig. 2). In regions with substantial vegetation changes, neglecting the feedback of BVOC emission caused by the BGP effects would introduce considerable uncertainty into an assessment of the environmental and climate effects of vegetation changes. Furthermore, the response of BVOC emission to the BGP effects adds complexity to assessing the aerosol climate effects from vegetation changes.

### Global diversity in biogeophysical modulation of BSOA and radiative effects

**Figure 3.**
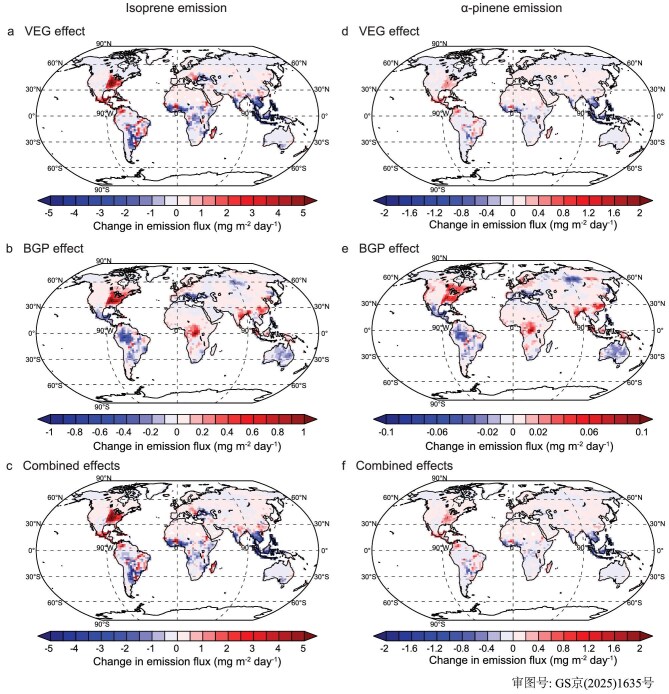
Global spatial patterns of projected BVOC emission changes (2000–2100, SSP245 scenario) induced by vegetation cover changes. Annual mean changes (mg m⁻² day⁻¹) for isoprene (ISOP; a–c) and α-pinene (APIN; d–f) emissions attributed to (a and d) vegetation-type change effect (VEG effect), (b and e) biogeophysical feedback effect (BGP), (c and f) combined net effect resulting from the sum of VEG and BGP effects.

The contrasting dominant feedback pathways and BGP effects identified in the US and EU regions can be generalized and extrapolated to global vegetation change studies. However, global vegetation changes present diverse pathways and complex spatial distributions, resulting in highly intricate biogeophysical feedback effects. The albedo and perturbation-dominated BGP effects discussed above on climate occur worldwide, influencing regional climate change. Moreover, the regional BGP effects induced by changes in vegetation further amplify climate impacts through their influence on atmospheric circulation, leading to even greater spatial heterogeneity in the climatic effects of global vegetation changes. From 2000 to 2100, the BGP effects of global vegetation changes induce alterations in various climate factors. Here, we focus on surface temperature and radiation flux changes, which influence BVOC emission the most. Besides the contrasting BGP and VEG effects on BVOC emission resulting from reforestation in the US and EU regions discussed above, central Africa experiences an increase in surface shortwave radiation and temperature due to vegetation changes, resulting in a notable rise in isoprene and α-pinene emissions (Fig. S14). In the central Amazon rainforest region, where vegetation changes in the future are minor (Fig. 1i), the BGP effects induced by surrounding vegetation changes lead to slight decreases in both surface temperature and surface downwards shortwave radiation (Fig. S14). Despite these modest changes, as one of the regions with the highest BVOC emission, even slight reductions in temperature and radiation lead to a large decrease in isoprene and α-pinene emissions (Fig. 3).

**Figure 4.**
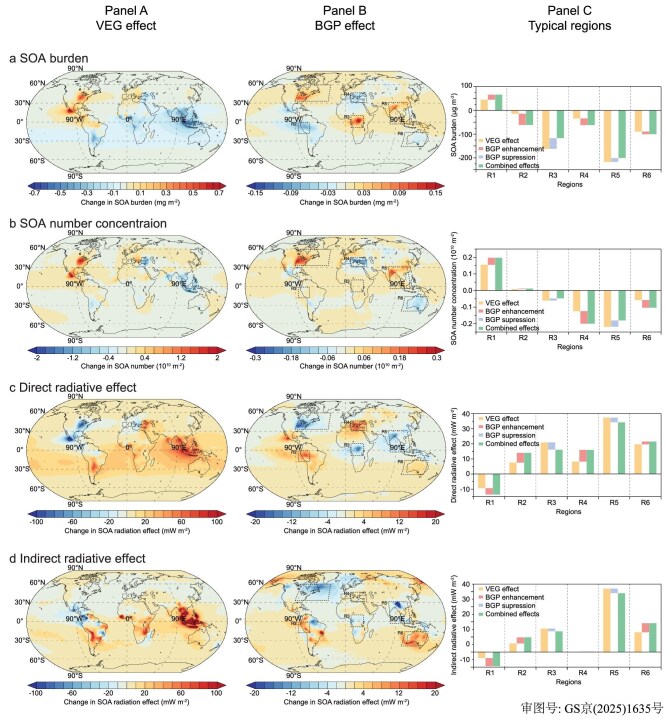
Projected global and regional changes in BSOAs and their radiative effects due to global vegetation changes in the future. Panel A shows global-scale changes attributed solely to the vegetation-type change effect (VEG effect) in BSOA burden (a; mg m⁻²), accumulation mode BSOA number concentration within the boundary layer (b; 10^10^ m⁻²), BSOA direct radiative effect (c; mW m⁻²), and BSOA indirect radiative effect (d; mW m⁻²). Panel B shows corresponding global-scale changes attributed solely to the biogeophysical feedback effect (BGP effect), with dashed boxes (R1–R6) indicating six typical regions analyzed in Panel C. Panel C presents regional changes for R1–R6 in the same four metrics, using bar charts, where yellow represents the contribution of the VEG effect, red represents enhancement of the VEG effect by the BGP effect, blue represents suppression of the VEG effect by the BGP effect, and green represents the combined effect (VEG + BGP).

From 2000 to 2100, the VEG effects alone lead to a global decrease of 20.88 Tg annually in isoprene emissions (Fig. 3a). Due to variations in vegetation changes, regions experiencing forest expansion (such as the eastern US and southern Mexico) show an increase in BVOC emission, while regions experiencing forest reduction (such as Southeast Asia and central Africa) exhibit a notable decrease in BVOC emission (Fig. 3a). However, the sign of the contribution from BGP effects of vegetation changes are not consistent, since they may either enhance or suppress changes in BVOC emission over the world, exhibiting spatial asymmetry in their effects (Fig. 3b). The BGP effects could enhance BVOC emission variations resulting from VEG effects in ∼52% of the global vegetation cover regions, leading to an average increase in isoprene emission of 18%. Meanwhile, in the remaining vegetation cover regions, emission variations resulting from VEG effects are suppressed by BGP effects, resulting in an average suppression of the emissions of isoprene by 14%. Similar spatial disparities are found in the BGP effects on the changes in emissions of α-pinene and BVOCs (Fig. 3e). The BGP effects alter the spatial pattern of BVOC emissions induced by global vegetation changes, thereby changing the pattern of BSOA burden and their number concentrations (Fig. 4a and b). This, in turn, alters the radiative effects of BSOAs (Fig. 4c and d), directly by scattering and absorbing solar radiation, and indirectly by modifying cloud properties through acting as cloud condensation nuclei (CCN), thereby exerting additional climate feedback.

Globally, some regions in North America, Europe and East Asia experience negative BSOA radiative effects from the VEG effects due to reforestation and afforestation, while others exhibit the opposite radiative effects (Fig. 4c and d). We selected six representative regions to discuss the BGP effects from changes in vegetation on BSOAs and its radiative effects. Region R1 stands out as the region with the most extensive reforestation and afforestation. The VEG effects lead to an average increase in BSOA burden of 45 μg m^−2^ (R1 region in Fig. 4a), an increase in accumulation mode BSOA number concentration of 0.16 × 10^10^ m^−2^ within the boundary layer (R1 region in Fig. 4b), resulting in a direct radiative effect of −9.4 mW m^−2^ (R1 region in Fig. 4c) and an indirect effect of −4.0 mW m^−2^ (R1 region in Fig. 4d). The BGP effects further enhance the increase in the BSOA burden by 47% in region R1 (R1 region in Fig. 4a), an increase in BSOA number concentration by 27% (R1 region in Fig. 4b), leading to a 45% enhancement in the direct radiative effect to −13.6 mW m^−2^ (R1 region in Fig. 4c) and a 132% enhancement in the indirect radiative effect to −9.3 mW m^−2^ (R1 region in Fig. 4d). In region R4, where forest cover increases (Fig. 1i), the BGP effects lead to a decrease in BSOAs (R4 region in Fig. 4a and b), weakening both the direct and indirect effects (R4 region in Fig. 4c and d). Region R2 experiences minimal vegetation changes (Fig. 1i), resulting in a decrease in BSOA burden of only 14.2 μg m^−2^ due to VEG effects (R2 region in Fig. 4a). However, the BGP effects exacerbate this reduction by a factor of 3.3 (R2 region in Fig. 4a), intensifying the direct radiative effect by 85% and the indirect radiative effect by 71% (R2 region in Fig. 4c and d). Regions R3 and R5 have a reduction in forest cover (Fig. 1i), leading to decreases in BSOA burden and number concentration due to VEG effects (R3 and R5 regions in Fig. 4a and b). However, the BGP effects offset 7%–28% of the decrease in BSOAs caused by vegetation changes (R3 and R5 regions in Fig. 4a and b), resulting in a reduction of ∼6 mW m^−2^ in the direct and indirect radiative effects (R3 and R5 regions in Fig. 4c and d). In region R6, although forest cover decreases, the BGP effects further reduce the change in BSOA burden and number concentration by 12% and 8% (R6 regions in Fig. 4a and b), respectively, intensifying the weakening of the direct radiative effect by 9.6% and the indirect radiative effect by 46% (R6 regions in Fig. 4c and d). Thus, the BGP effects introduce complex asymmetric changes in BSOAs and their radiation effects resulting from vegetation changes, leading to increased spatial heterogeneity of atmospheric environmental and climate change.

## CONCLUSIONS

By conducting a series of numerical sensitivity experiments under future scenarios involving changes in vegetation, and the resultant changes in climate and emissions of BVOCs, we find that climate change induced by the BGP effects of reforestation and afforestation can alter emissions of BVOCs, affecting BSOA concentrations and their radiative cooling effects. However, the impact of this biogeophysical feedback may either amplify or offset the enhancement in BSOAs and the cooling effect of aerosols caused by reforestation and afforestation. When reduced surface albedo due to reforestation and afforestation dominates the BGP effects, it increases absorption of solar radiation, leading to local warming. Higher temperatures enhance BVOC emissions from vegetation, which promotes BSOA formation and amplifies its cooling effect through enhanced aerosol scattering and cloud interactions. When increased evapotranspiration and associated cloud formation due to airflow perturbation induced by reforestation and afforestation dominate, the enhanced upward moisture flux reduces surface solar radiation via increased cloud cover. This suppresses both temperature and BVOC emissions, thereby diminishing BSOA production and partially offsetting its potential cooling effect. The net climate impact of vegetation changes depends on which feedback pathway prevails in a given region.

Previous studies have identified model-dependent uncertainties in Earth system models when simulating biogeophysical feedback caused by vegetation changes, leading to divergent quantitative assessments [44]. The largest uncertainty arises from the overestimation of albedo during snow-covered periods [45]. In this study, however, BVOC emissions and subsequent BSOA formation primarily occur in warm seasons, as emission intensity decreases sharply with temperature. Thus, uncertainties in albedo simulation during cold seasons (e.g. snow-covered periods) likely have minimal influence on our BVOC and BSOA response assessments. Furthermore, our findings reveal that the biogeophysical feedback effects of vegetation changes exhibit highly complex spatial, seasonal and climatic dependencies, as well as strong sensitivity to underlying surface characteristics. This suggests that quantifying this feedback requires case-specific modeling under given environmental conditions. The key takeaway of our study is to highlight that vegetation changes can trigger opposing biogeophysical feedback pathways, which in turn exert contrasting radiative effects via BSOA formation. This mechanism must be carefully considered in climate effect predictions and assessments, particularly in regions undergoing significant vegetation changes. Additionally, increased aerosols can influence the albedo-driven warming effect induced by vegetation changes by reducing surface radiation, as well as affect vegetation growth by altering temperature and precipitation patterns, thereby further modulating the BGP effects of vegetation changes [46]. These feedback mechanisms were not considered in our study. However, since the BSOA changes driven by vegetation variation account for less than 10% of the anthropogenic aerosol increase since industrialization, we infer that the feedback effect of vegetation-induced BSOA changes on BGP effects is likely negligible in most regions globally. Moreover, plant respiration is modulated by climate change, which may further contribute to the uncertainty in estimating the net climatic effects of vegetation dynamics [47].

The BGP effects of future global vegetation changes have specific feedback impacts on regional BSOA radiative effects, depending on regional vegetation and climate conditions. This leads to varied climate effects of global vegetation changes across different regions. Therefore, when assessing the future climate effects of regional vegetation changes such as reforestation and afforestation, it is crucial to consider the response of local and teleconnected regional BSOA radiative effects to the BGP effects of vegetation changes [48]. This comprehensive mechanism will more effectively guide the selection of regions for human-induced vegetation changes such as reforestation and afforestation to maximize climate change mitigation. Given that reforestation in albedo-dominated regions (e.g. eastern US) amplifies BSOA cooling through BGP effects, it is recommended that such areas prioritize planting high-BVOC-emitting species (e.g. broadleaf forests) to enhance aerosol-mediated cooling. Conversely, in perturbation-dominated regions (e.g. Western Europe), where reforestation-induced BGP effects suppress BSOA formation, implementing albedo-optimization measures (e.g. mixed-species planting) is essential to balance radiation budgets. Consequently, optimizing reforestation for climate mitigation necessitates diagnosing regional dominant feedback mechanisms before implementation, then tailoring species selection and forest structure to either amplify BVOC-mediated aerosol cooling or counter radiative trade-offs, thereby maximizing co-benefits for carbon sequestration and local climate adaptation. Although the net climatic regulation effect of BVOC emission changes induced by vegetation shifts cannot be overlooked, the overall climate impact of vegetation change must be comprehensively assessed in conjunction with biogeophysical processes and carbon cycle effects.

## METHODS

### Model description

The Community Earth System Model (CESM) version 1.2.2, integrated with the University of Michigan's Integrated Massively Parallel Atmospheric Chemical Transport (IMPACT) aerosol model, was utilized for this study. In this setup, the IMPACT aerosol module uses meteorological data from CESM at each time step, with no feedback on CESM from aerosol changes within IMPACT. This model combines modal and sectional approaches to simulate 15 aerosol species, including sulfate (in nucleation, Aitken and accumulation modes), soot from biomass burning (bSoot), soot from fossil fuel and biofuel burning (fSoot), dust and sea salt. Dust and sea-salt aerosols are distributed across four bins with varying radii, and non-sulfate aerosols are internally mixed with sulfate and secondary organic aerosols (BSOAs) through condensation and coagulation processes. The detailed model description and setup can be found in a previous study [49]. Anthropogenic aerosol precursors, along with black and organic carbon emissions for the year 2000, were sourced from IPCC datasets and held constant throughout the study. Biogenic secondary organic aerosol precursors, such as isoprene and α-pinene, were estimated using the MEGAN model coupled to CESM [38,50]. For the simulation of BSOAs, four gaseous precursors were considered: isoprene, α-pinene, limonene and aromatics [51]. The model employed an explicit gas-phase chemical mechanism to predict the formation of semi-volatile organic compounds (SVOCs) from precursor oxidation by ozone and hydroxyl radicals [52]. Additionally, aqueous-phase reactions of glyoxal and methylglyoxal, along with heterogeneous reactions of isoprene epoxydiols (IEPOX), were incorporated to generate BSOAs [53]. These processes result in the formation of low-volatility products and thermodynamic partitioning followed by oligomerization within aerosols. Biogenic secondary organic aerosols from aqueous-phase processes are distributed to particles acting as CCN, and all newly formed BSOA are internally mixed with sulfate, bSoot, fSoot, dust and sea salt according to its formation mechanism [54]. Low-volatility highly oxygenated molecules from α-pinene oxidation were used to drive organic particle nucleation, which are generated through explicit gas-phase and particle-phase reactions [55]. The model includes heteromolecular nucleation of sulfuric acid and organics, neutral organic nucleation and ion-induced organic nucleation, with rates parameterized from experimental results [55]. New particles resulting from organic nucleation (newBSOAs) can grow to Aitken and accumulation modes through coagulation and condensation processes, covering the same size ranges as new sulfate particles [49].

To compute aerosol optical properties and cloud droplet activation, an offline radiative transfer model was employed. This enabled the separate estimation of direct and indirect radiative effects of aerosols. Aerosol concentration, number and mixing state were derived from CESM/IMPACT model calculations. Internally mixed aerosols were categorized into six types based on their formation mechanisms, with refractive indices and hygroscopicity determined by volume-weighting of individual species. Köhler theory was applied to predict water uptake by aerosols, impacting their size and refractive indices. Comprehensive model specifications and details are provided in previous publications [54,56]. The model's capability in simulating BSOA and aerosol optical depth has been comprehensively evaluated against extensive ground-based observations, aircraft measurements and satellite remote sensing data in our previous studies, demonstrating its reliability in simulating BSOA and related radiative effects [49,52,53,55,57].

### Simulation scenarios

To investigate the impact of vegetation changes on emissions of BVOCs, BSOAs and climate change, we set up three vegetation scenarios based on the SSP245 scenario: global vegetation scenarios for the years 2000 (20V) and 2100 (21V), and a sensitivity scenario (SenV) that only considers reforestation in the US and EU from 2000 to 2100 (regions as shown by dashed boxes in Fig. 1i). The SenV scenario aims to study the BGP effects and feedback of regional vegetation changes clearly, avoiding interference from meteorological changes due to vegetation changes in other regions. The 21V scenario is used to examine the complex effects of global vegetation changes under the SSP245 scenario.

Each of the three vegetation scenarios was used to drive the CESM model to simulate the distribution of global climate changes influenced by the BGP effects of vegetation changes. These were named 20M, 21M and SenM for years 2000 and 2100 as well as the sensitivity scenario, respectively. The difference between 21M and 20M represents the BGP effects on climate change due to changes in vegetation from 2000 to 2100, while the difference between SenM and 20M represents the regional BGP effects due to reforestation in the US and EU regions. Each scenario was simulated for 50 years.

Next, using the CESM’s emissions of BVOCs simulation module combined with the three meteorological scenarios, we calculated the emissions of BVOCs under each vegetation scenario for 50 years. We then calculated the emissions of BVOCs for five cases based on the combinations of meteorological scenarios and vegetation scenarios, which were named 20V20M, 21V20M, SenV20M, 21V21M and SenVSenM. Using the 50-year average emissions of BVOCs calculated from these cases, we employed the CESM/IMPACT model to simulate the distribution of global BSOA concentrations under these five scenarios for 5 years. Additionally, an offline radiative transfer model was used to calculate the direct and indirect radiative effects of 5-year average BSOAs for each case. All cases were concluded in Table S1.

The differences between the 21V20M and SenV20M cases and the 20V20M case indicate the effects of vegetation-type changes on emissions of BVOCs, BSOA formation, and radiative effects (VEG effects) as a result of the vegetation change. The differences between the 21V21M and 21V20M cases, as well as between the SenVSenM and SenV20M cases, indicate the effects of climate change, driven by the biogeophysical impacts of vegetation changes, on emissions of BVOCs, BSOA formation, and radiative effects (BGP effects). The representations of difference between cases are concluded in Table S2.

## Supplementary Material

nwaf323_Supplemental_File
