# Supplementary material for: Reforestation-induced aerosol cooling effects divergently modulated by various types of biogeophysical feedback
Source: Natl Sci Rev. 2025 Aug 15;12(10):nwaf323. doi: 10.1093/nsr/nwaf323 (PMC12449073; doi:10.1093/nsr/nwaf323)
Supplement: nwaf323_Supplemental_File [file nwaf323_supplemental_file.pdf]

Supplementary Materials for
Reforestation-induced aerosol cooling effects divergently modulated by various biogeophysical feedbacks

Jialei Zhu[1], Joyce E. Penner[2], Hao Liu[1], Qinghao Guo[1], Yaxin Liu[1], Junjun Deng[1], Xi Zhao[1], Cong-Qiang Liu[1], Pingqing Fu[1*]

[1] Institute of Surface-Earth System Science, School of Earth System Science, Tianjin University, Tianjin, China.

[2] Department of Climate and Space Sciences and Engineering, University of Michigan, Ann Arbor, MI, USA.

*Correspondence to: Pingqing Fu (fupingqing@tju.edu.cn)

This PDF file includes:

Supplementary Figures S1–S14

Supplementary Tables S1–S2

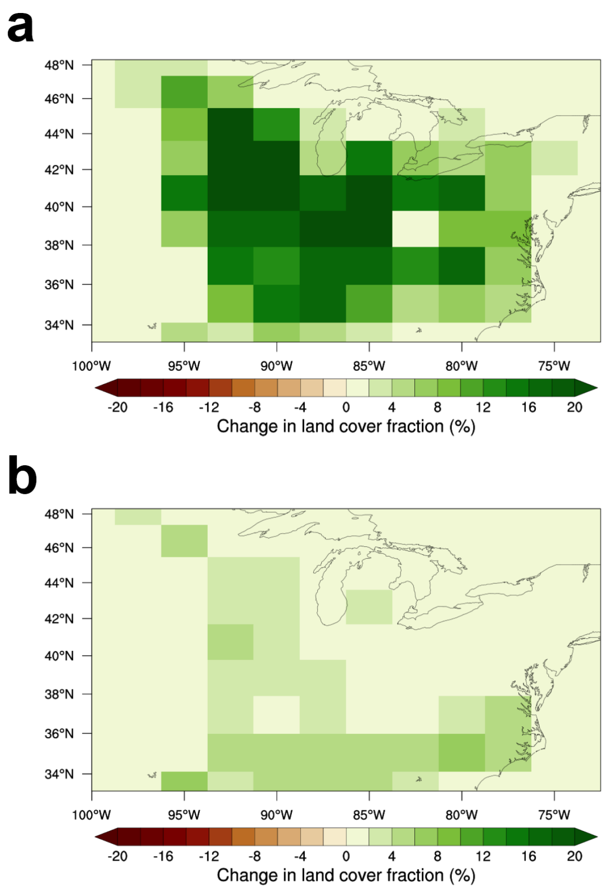

Supplementary Figure 1. Changes in the cover fraction of deciduous broadleaf forests (a) and evergreen needleleaf forests (b) in the US region from 2000 to 2100 based on SSP245 scenario

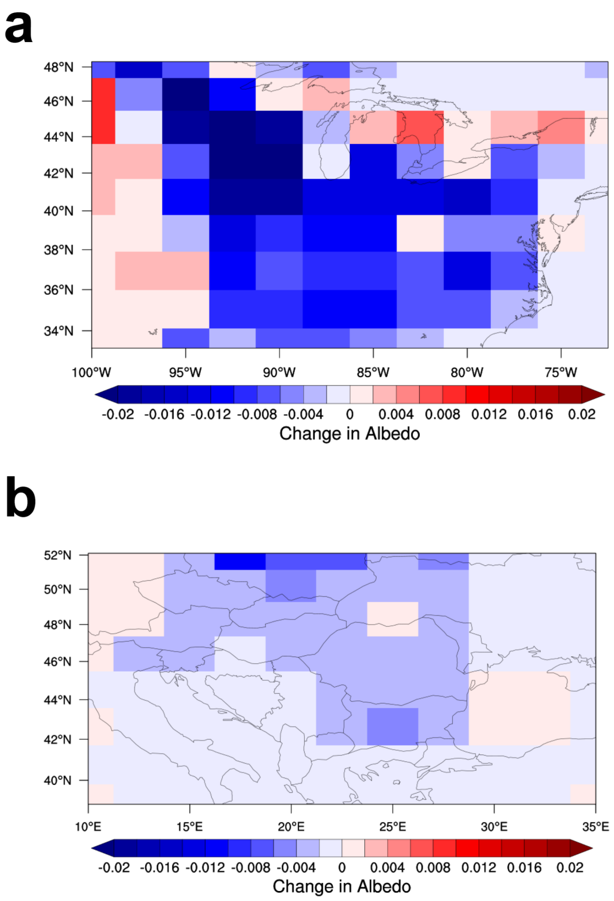

Supplementary Figure 2. Changes in albedo due to reforestation from 2000 to 2100 in the US (a) and EU (b) regions.

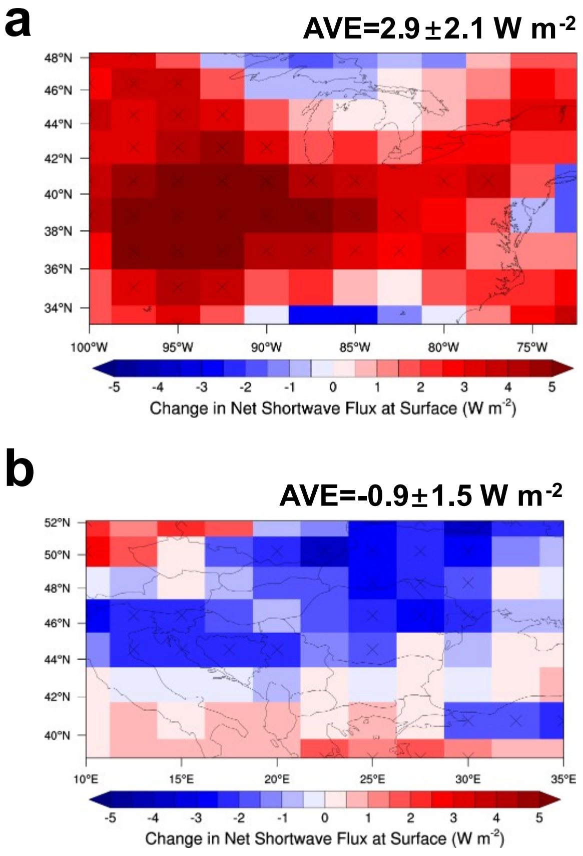

Supplementary Figure 3. Changes in the average surface net shortwave radiation flux (W m^{-2}) in the summer (June, July and August) due to the biogeophysical effect of reforestation from 2000 to 2100 in the US (a) and EU (b) regions. Differences significant at the 90% level according to a Student's t test are depicted by crosses.

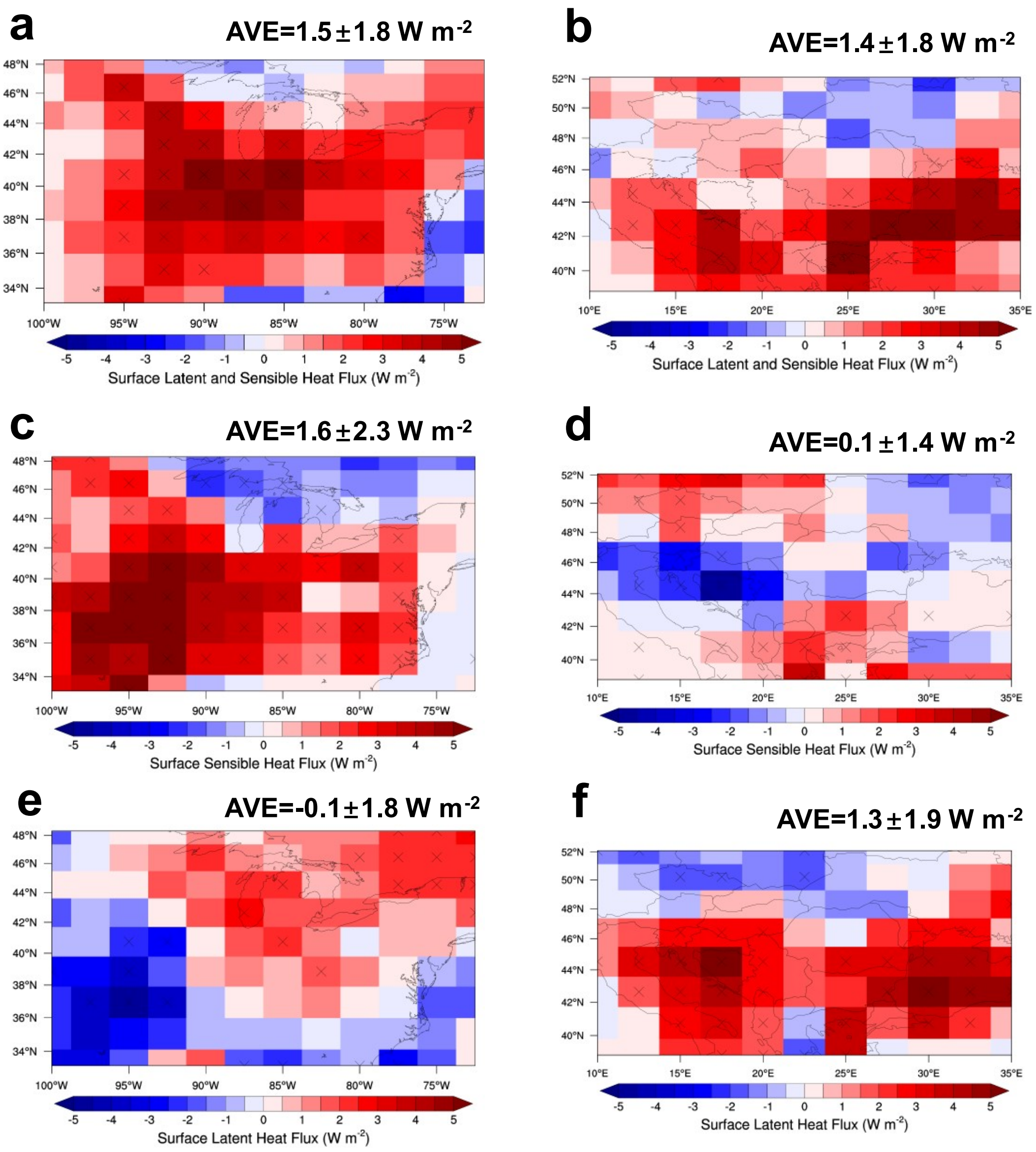

Supplementary Figure 4. Changes in the total of surface latent and sensible heat flux (a, b, W m^{-2}), surface sensible heat flux (c, d, W m^{-2}) and surface latent heat flux (e, f, W m^{-2}) in the summer (June, July and August) due to the biogeophysical effect of reforestation from 2000 to 2100 in the US (a, c, e) and EU (b, d, f) regions. Differences significant at the 90% level according to a Student's t test are depicted by crosses.

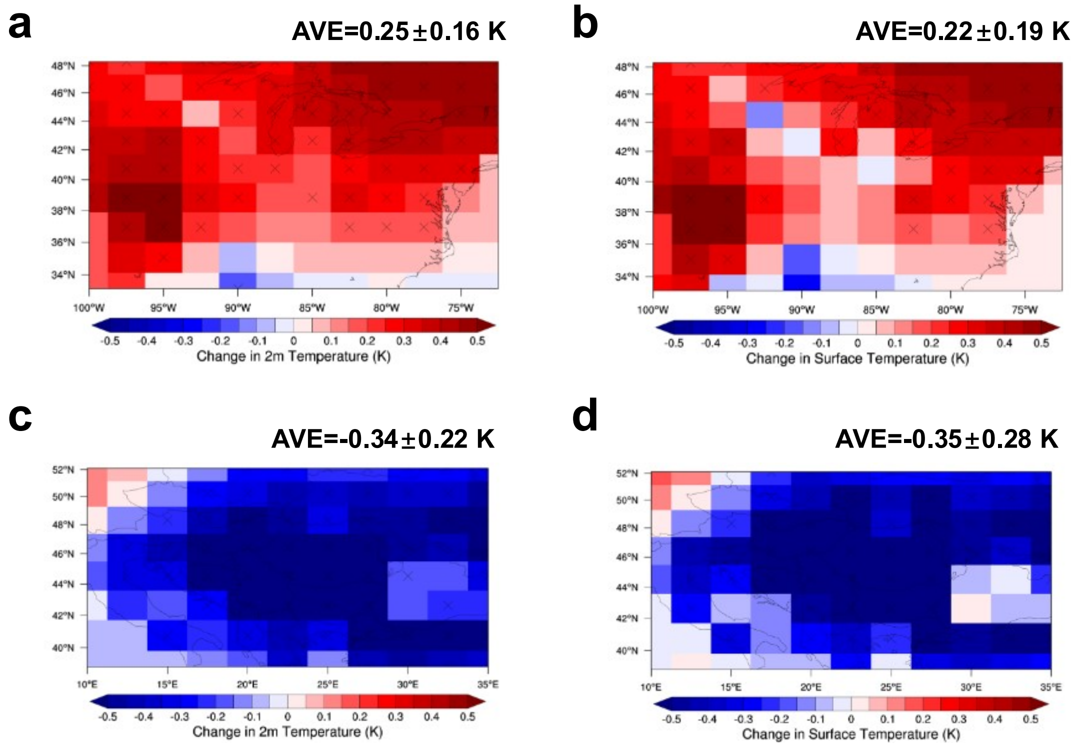

Supplementary Figure 5. Changes in the 2m (a, c) and surface (b, d) air temperature (K) in the summer (June, July and August) due to the biogeophysical effect of reforestation from 2000 to 2100 in the US (a) and EU (b) regions. Differences significant at the 90% level according to a Student's t test are depicted by crosses.

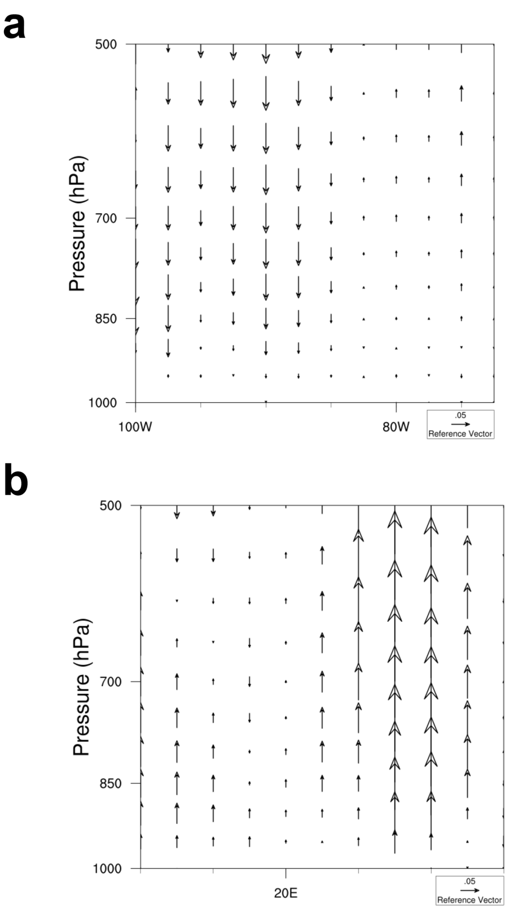

Supplementary Figure 6. Changes in the vertical wind in the summer (June, July and August) due to the biogeophysical effects of reforestation from 2000 to 2100 in the US (a) and EU (b) regions.

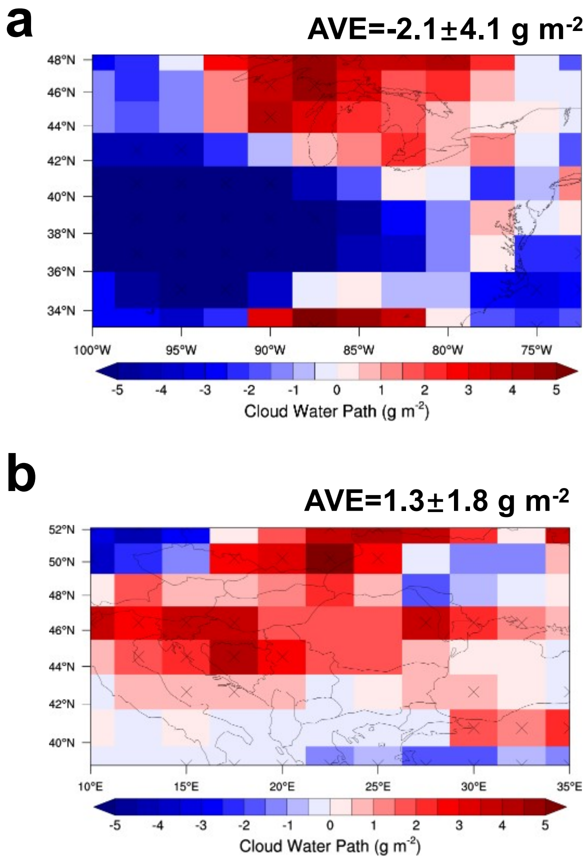

Supplementary Figure 7. Changes in total cloud water path (g m^{-2}) in the summer (June, July and August) due to the biogeophysical effect of reforestation from 2000 to 2100 in the US (a) and EU (b) regions. Differences significant at the 90% level according to a Student's t test are depicted by crosses.

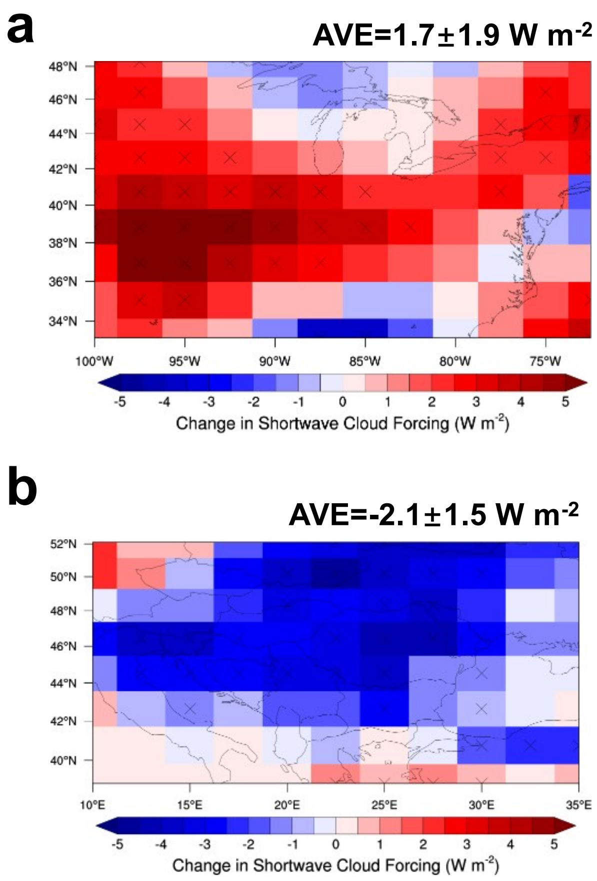

Supplementary Figure 8. Changes in cloud shortwave radiative forcing (W m^{-2}) in the summer (June, July and August) due to the biogeophysical effect of reforestation from 2000 to 2100 in the US (a) and EU (b) regions. Differences significant at the 90% level according to a Student's t test are depicted by crosses.

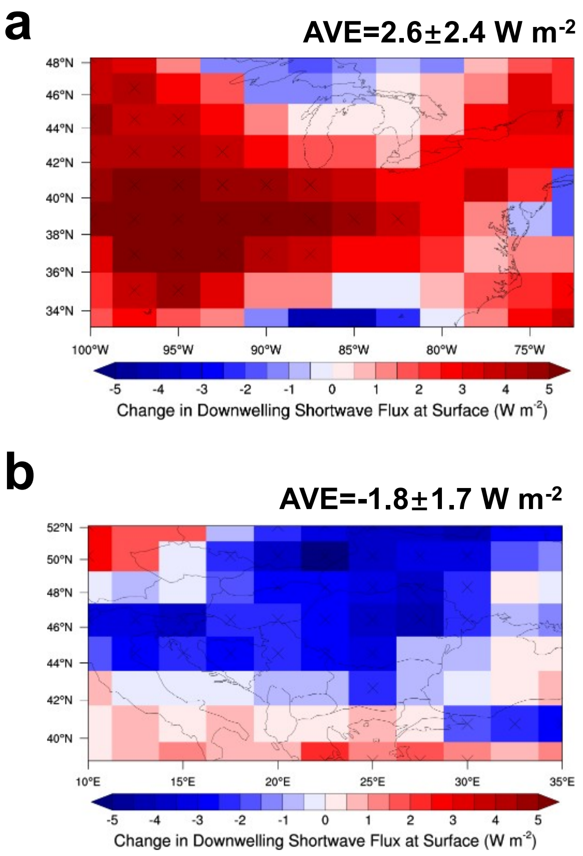

Supplementary Figure 9. Changes in downwelling shortwave flux at surface (W m^{-2}) in the summer (June, July and August) due to the biogeophysical effect of reforestation from 2000 to 2100 in the US (a) and EU (b) regions. Differences significant at the 90% level according to a Student's t test are depicted by crosses.

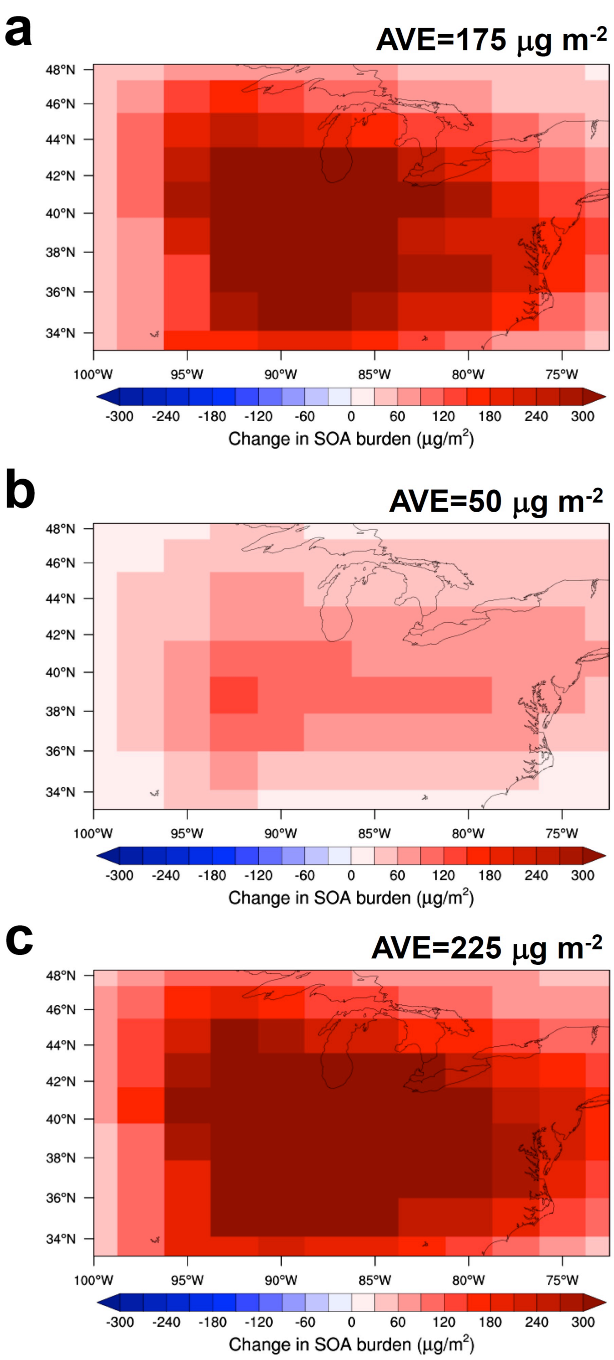

Supplementary Figure 10. Changes in annual average SOA burden (μg m^{-2}) due to the vegetation change (VEG effects, a), biogeophysical feedbacks (BGP effects, b) and their combined effects (c) induced by reforestation from 2000 to 2100 in the US region.

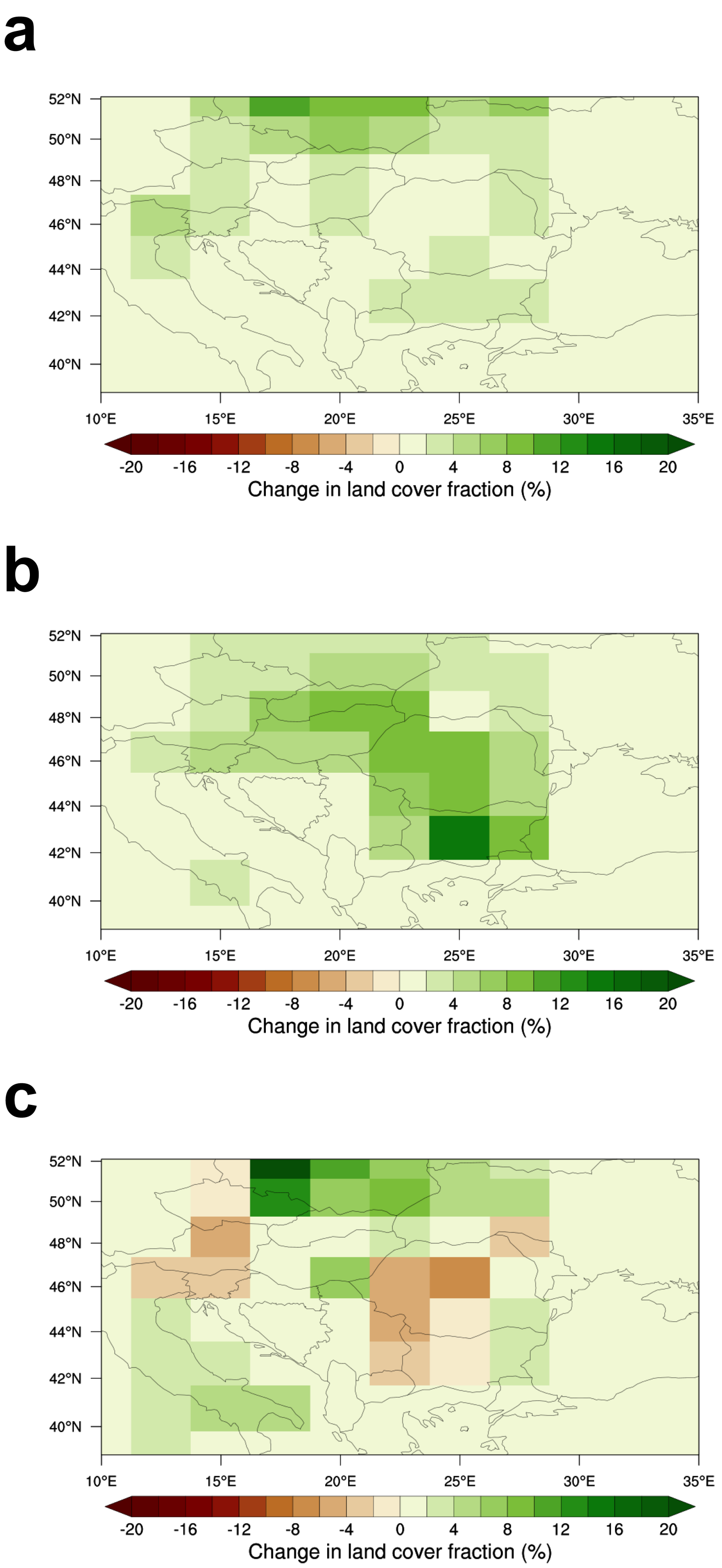

Supplementary Figure 11. Changes in the cover fraction of evergreen needleleaf forests (a), deciduous broadleaf forests (b) and grasslands (c) in the EU region from 2000 to 2100 based on SSP 245 scenario

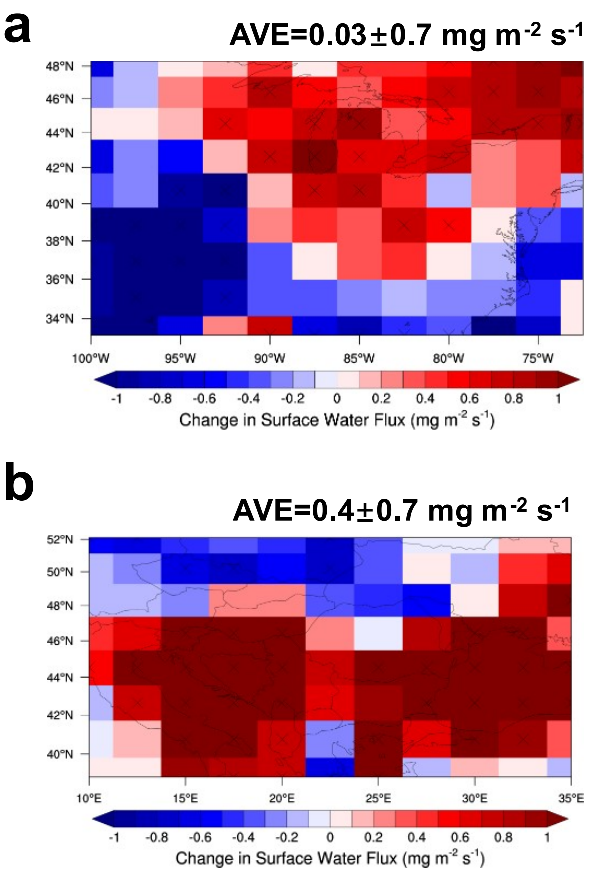

Supplementary Figure 12. Changes in upward water vapor flux (mg m^{-2} s^{-1}) in the summer (June, July and August) due to the biogeophysical effect of reforestation from 2000 to 2100 in the US (a) and EU (b) regions. Differences significant at the 90% level according to a Student's t test are depicted by crosses.

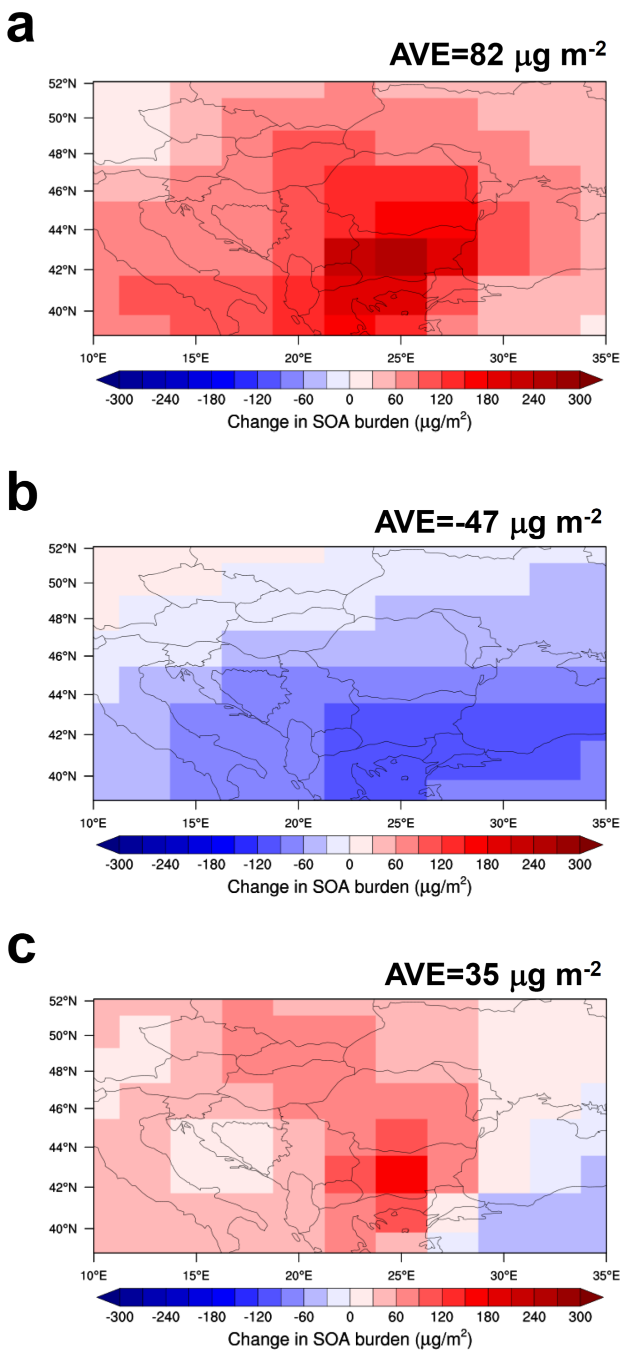

Supplementary Figure 13. Changes in annual average SOA burden (μg m^{-2}) due to the vegetation cover change (VEG effects, a), biogeophysical feedbacks (BGP effects, b) and their combined effects (c) induced by reforestation from 2000 to 2100 in the EU region.

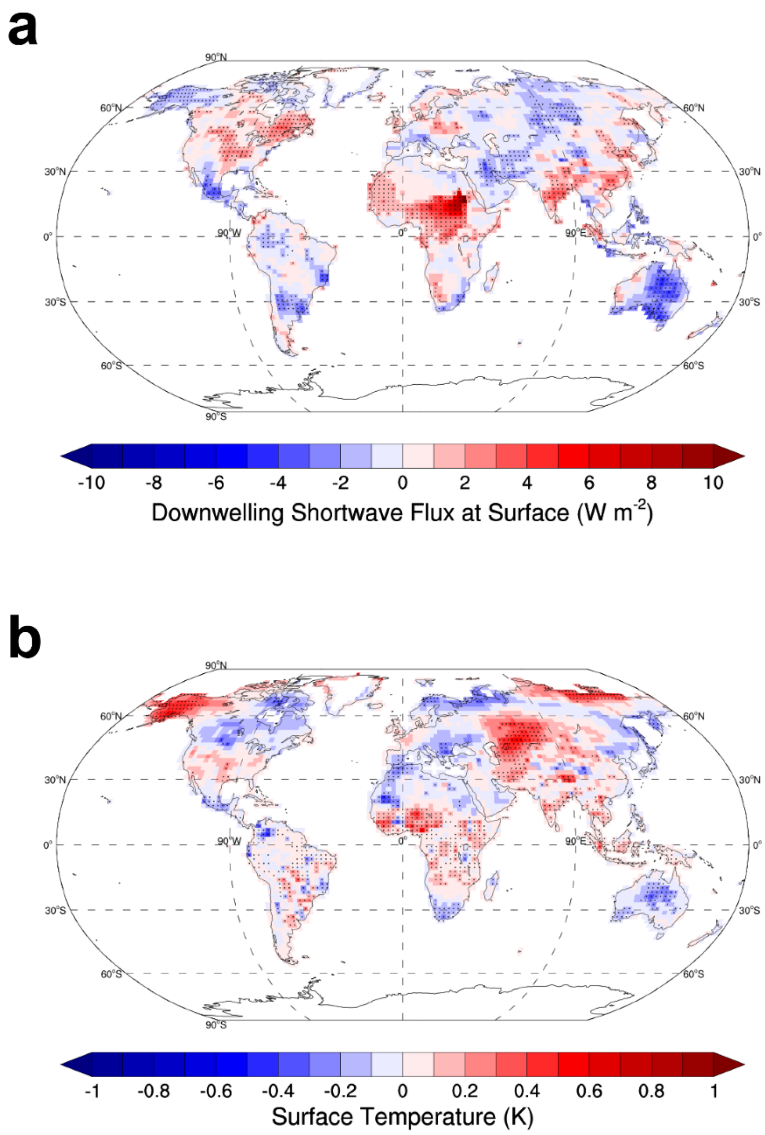

Supplementary Figure 14. Changes in annual average downwelling shortwave flux at surface (a, unit: W m⁻²) and 2m air temperature (K) due to the biogeophysical effect of global vegetation cover change from 2000 to 2100 based on SSP245 scenario. Differences significant at the 90% level according to a Student's t test are depicted by points.

Supplementary Table 1 Description of simulation cases

No.	Cases	Vegetation scenario	Meteorological scenario
1	20V20M	Present day	Present day
2	21V20M	Change globally	Present day
3	SenV20M	Change only in US/EU	Present day
4	21V21M	Change globally	Change globally
5	SenVSenM	Change only in US/EU	Change only in US/EU

Supplementary Table 2 Representation of difference between cases

Difference between cases	Representation
21V20M-20V20M	Global VEG effect
SenV20M-20V20M	US/EU regional VEG effect
21V21M-21V20M	Global BGP effect
SenVSenM-SenV20M	US/EU regional BGP effect
21V21M-20V20M	Global VEG+BGP effect
SenVSenM-20V20M	US/EU regional VEG+BGP effect
